# Effectiveness of a Training Course for General Practice Nurses in Motivation Support in Type 2 Diabetes Care: A Cluster-Randomised Trial

**DOI:** 10.1371/journal.pone.0096683

**Published:** 2014-05-05

**Authors:** Lise Juul, Helle T. Maindal, Vibeke Zoffmann, Morten Frydenberg, Annelli Sandbaek

**Affiliations:** 1 Department of Public Health, Section for General Practice, Aarhus University, Aarhus, Denmark; 2 Department of Public Health, Section for Health Promotion and Health Services, Aarhus, University, Aarhus, Denmark; 3 Steno Diabetes Centre, Gentofte, Denmark; 4 Nasjonal kompetansetjeneste for læring og mestring innen helse (NKLMH), Oslo University Hospital, Oslo, Norway; 5 Department of Public Health, Section for Biostatistics, Aarhus University, Aarhus, Denmark; Iran University of Medical Sciences, Iran (Republic of Islamic)

## Abstract

**Background:**

Type 2 diabetes is a common metabolic disease with the potential for prevention of complications. The prevention requires a high level of lasting actions from the patients, which may be burdensome. The aim of this trial was to evaluate the effectiveness of a training course for general practice nurses in motivation support at 18 months follow-up in the affiliated type 2 diabetes population.

**Methods:**

Forty general practices with nurse-led diabetes consultations from the area of Aarhus, Denmark were randomised 1∶1 to either intervention or usual practice. Intervention practices were offered a 16-hour Self-determination theory - based course including communication training for general practice nurses delivered over 10 months. The affiliated diabetes populations (aged 40–74 years) were identified from registers (intervention n = 2,005; usual n = 2,029). Primary outcomes were register-based glycated haemoglobin (HbA_1c_) -, total cholesterol levels, and well-being measured by the Problem Areas In Diabetes scale (PAID) and the mental component summary score, SF12 (SF12, mcs). Intention-to-treat analyses were performed. Predefined subgroups analyses were performed.

**Results:**

The differences between the intervention- and the control practices’ mean HbA_1c_ and total cholesterol at follow-up adjusted for baseline values and clustering were respectively: −0.02%-points (95% CI: −0.11 to 0.07; p: 0.67); 0.08 mmol/l (95% CI: 0.01 to 0.15; p: 0.02). Differences in median scores adjusted for clustering were for PAID: 1.25; p = 0.31 and SF12, mcs: 0.99; p = 0.15. Women in intervention practices differed from women in usual practices on mean HbA_1c_: −0.12%-points (−0.23 to −0.02; p = 0.02) and SF12, mcs: 2.6; p = 0.01.

**Conclusions:**

Offering a training course for general practice nurses in applying the Self-determination theory in current type 2 diabetes care had no effect compared with usual practice measured by HbA_1c_ and total cholesterol levels and the well-being at 18 months of follow-up in a comprehensive register-based diabetes population. Subgroup analyses suggested a possible effect in women, which deserves further attention.

**Trial Registration:**

ClinicalTrials.gov (Identifier NCT01187069).

## Background

Type 2 diabetes mellitus is a common metabolic disease with profound consequences such as visual impairment, renal failure, neuropathy with risk of amputation, myocardial infarction, stroke and increased mortality [1]–[3]. Evidence on the prevention of these complications suggest both poly-pharmacological treatment including glucose-lowering, lipid-lowering and blood pressure lowering treatment and health behaviour including non-smoking, healthy diet and physical activity [4]. The prevention of the complications requires a high level of lasting actions from the patients, which may be perceived as demanding by the patients, and lead to emotional distress. How health care providers support the patients living with the chronic disease is therefore essential.

In Danish healthcare, as in many other countries, type 2 diabetes care is provided by general practice in the framework of one annual 30 minute – and three quarterly 15 minutes consultations. In 2009, a growing share of the diabetes care was delegated from general practitioners to nurses employed in the practices (GP nurses) [5]. We therefore established a training course aiming to improve the contents in the nurse-led diabetes consultations within the framework of current type 2 diabetes care and for the benefits of the patients with type 2 diabetes. In the development of the training course, we addressed the huge challenge it is for health care providers to adhere to the treatment guidelines and also support the patients’ motivation for the recommendations, and support their well-being. Self-determination Theory (SDT) was chosen as the underlying theory for our intervention [6]. SDT proposes both a patient-centred approach with the main focus on the underlying reasons for motivation for actions, and a high emphasis on the importance of adequate information from the health care providers. SDT was well supported by observational studies where core elements of the theory; autonomy support and autonomous motivation were found associated with higher quality of life and better clinical outcomes in patients with diabetes [7]–[9]. Furthermore, an explanatory trial in a Danish diabetes outpatient clinic supported the theory [10]; but, documentation of the effect of applying SDT in current type 2 diabetes care in general practice was lacking. The aim of the present trial was to evaluate the effectiveness of a training course for GP nurses in applying SDT in current type 2 diabetes care on glycated haemoglobin (HbA_1c_)-, total cholesterol levels and well-being in the diabetes population at an 18-month follow-up. We expected that the intervention would enhance autonomous motivation in the patients with type 2 diabetes; which would improve their well-being and maintain their motivation for health behaviour changes; which again would reduce their risk profile regarding complications indicated by HbA_1c_- and total cholesterol levels. We expected that the patients with type 2 diabetes regardless of education level, gender or age would benefit from the intervention.

## Methods

### Design, Setting and Study Population

A cluster-randomised pragmatic trial including 40 general practices with nurse-led type 2 diabetes consultations was conducted in the area of Aarhus, Denmark. The median number of nurses in the included practices was 2 (range 1–5). A detailed description of the intervention, the context and the recruitment process has previously been published [11]. The protocol for this trial and supporting CONSORT checklists are available as supporting information; see Protocol S1 and Checklist S1 + S2.

The 40 general practices were randomised 1∶1 to either intervention or usual practice by a statistician who was blinded to the identity of the practices.

**Figure 1:**
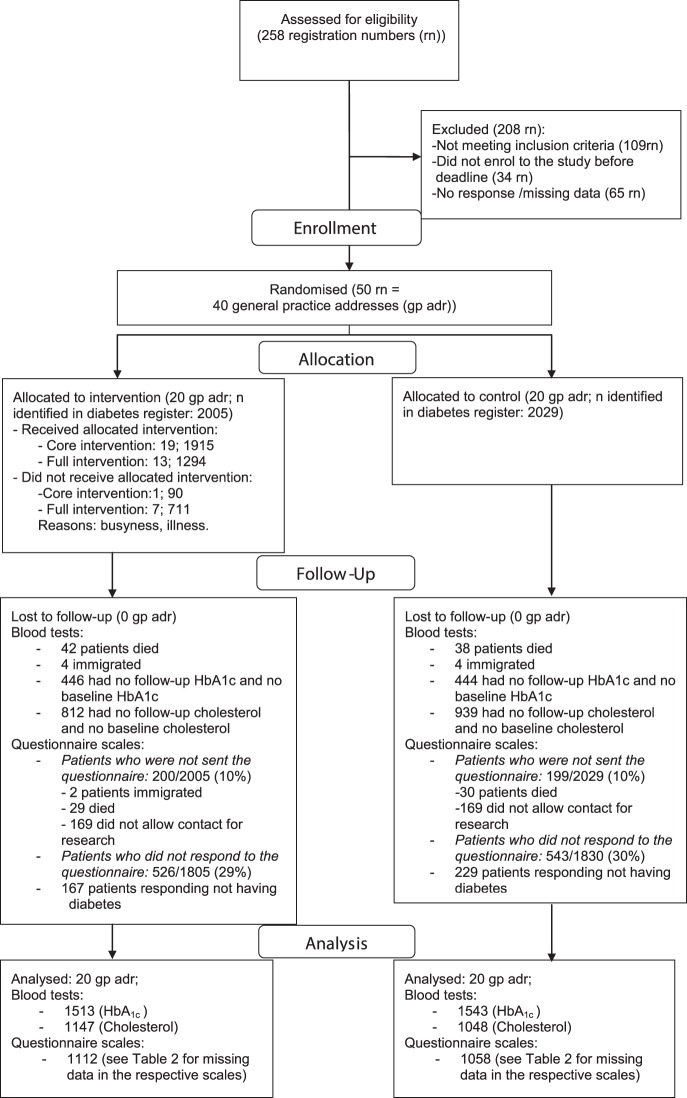
Flowchart. The inclusion of general practices and the associated diabetes populations in a trial evaluating the effectiveness of a training course for general practice nurses in applying Self-determination theory in current type 2 diabetes care, DK 2011.

The diabetes population aged 40–74 years in October 2009 and listed with the included practices in January 2011 (n = 4,034) was identified in the Central Denmark Region’s Chronic Disease Database where patients with diabetes were identified by an algorithm based on health registers. We excluded 61 patients, 59 of whom died and 2 emigrated between October 2009 and May 2011. A total of 338 patients did not wish to be contacted for research reasons during the spring of 2011. This left 3635 patients for the study of self-reported outcomes (Figure 1). Another 27 patients were excluded in the analyses based on the blood tests; 21 died and 6 emigrated between May 2011 and September 2011; this left 3946 for the register-based study of clinical outcomes.

### Intervention

The intervention practices were offered a 16-hour course with interactive training targeted the nurses employed in the practices. The core course was delivered as two coherent afternoons (month 0). Furthermore; in two following single afternoons (months 2 and 5), and a 30-minute visit to the practice by one of the course teachers (month 10), during which implementation issues were addressed. The course was designed to meet the SDT-based recommendations on health care provider behaviour [12], [13], and it included the following themes: 1) Patient - health care provider relationships, 2) Communication skills, 3) Patient worksheets, 4) Current treatment recommendations of type 2 diabetes, and 5) Implementation of the course content in daily practice. The theoretical framework of these themes has previously been elaborated [11].

Usual practices were randomly drawn from among intervention practice applicants and were informed by letter about their status as usual practice.

### The Outcomes

The primary outcomes were HbA_1c_ and total cholesterol levels, and well-being, which were measured by the Problem Areas In Diabetes (PAID) scale [14] and the mental component summary score from the SF-12 (SF12, mcs) [15]. HbA1c and total cholesterol are frequently used outcome measures as proxy indicators for prognosis in diabetes [16]. HbA1c is a measure of the average plasma glucose concentration over the past two months. Total cholesterol is a measure of the level of lipids in the blood, and an elevated concentration is a risk factor for cardiovascular diseases.

PAID is a validated 20-item measure of perceived burden of living with diabetes [14], [17]–[19]. Questionnaire responses were made on a 5-point scale from 0 (not a problem) to 4 (serious problem). The scores were calculated as the sum of the 20 items multiplied by 1.25 with higher scores indicating higher levels of emotional burden living with diabetes. The SF12, mcs was calculated after standardised procedures with higher scores indicating greater psychological well-being.

Secondary motivation outcomes were assessed by validated SDT-based questionnaires [7]–[9], [20], [21], which had been translated into Danish according to a standardised procedure [10]. Perceived autonomy support was measured by the 6-item Health Care Climate Questionnaire (HCCQ), degrees of controlled and autonomous motivation were measured by the 19-item Treatment Self-Regulation Questionnaire (TSRQ) and perceived competence regarding living with diabetes was measured by the 4-item Perceived Competence for Diabetes Scale (PCDS) questionnaire. Responses to the SDT questionnaires were made on a 7-point scale from 1 (strongly disagree) to 7 (strongly agree). The scores were calculated as the average of the items for each individual scale. Higher average scores represent higher levels of perceived autonomy support, controlled motivation, autonomous motivation, and perceived competence in diabetes [22].

Cronbach’s α s of the used scales corresponded to previous Danish results [10], [23]. The HCCQ scale, the autonomous TSRQ scales and the PCDS had a skewed distribution for all items showing high ceiling effects (27–39% responded strongly agree).

### Data

Data on HbA_1c_ and cholesterol measurements were retrieved from the Region’s laboratory database and the self-reported patient data were obtained in a larger survey “Life with Diabetes”; a mailed questionnaire which in addition to being sent to the included diabetes population 16 months after the core course, also was sent to a random sample of 9960 people from the Central Denmark Region’s Chronic Disease Database. Two reminders were sent three weeks apart, and the questionnaires were completed 16–18 months after the core intervention. The register-based blood test data and the questionnaires were pooled by using the unique civil registry number assigned to all Danish citizens. Furthermore, the data were linked to social data (education, ethnicity, cohabitation) from Statistics Denmark. The following categorisation of data was used; educational level: ≤10 years of education or >10 years of education, ethnicity: Danish background or immigrant, cohabitation: living together with spouse/partner or living alone, and age: <60 years or ≥60 years.

### Process Data

Eleven months after the core intervention, an evaluation questionnaire was sent to all the nurses who partly or fully completed the course. This questionnaire contained questions on the nurses’ self-reported behavioural changes regarding autonomy support. They were asked to score their perceived autonomy supportive competences [12], [13] on a scale from 1–10 (1 = rarely, 10 = always) before the course and 11 months after the course. Further, they were asked about self-perceived, important behavioural changes, to which extent they had used the presented tools, and which part of the course they found most advantageous.

Further process data were collected; 1) The patient questionnaire asked questions about whether the patients had participated in a nurse-led diabetes consultation during the follow-up time to ascertain the potential reach of the intervention, and 2) the patients’ perception of autonomy support from the nurses was measured by the HCCQ in the patient questionnaire.

### Statistical Analysis

Analyses were performed according to the intention-to-treat principle. HbA_1c_ and total cholesterol baseline values were the average of the values measured within the last 12 months before course start. The follow-up values were the average of the values measured during the “observational window” 15–21 months after the core intervention. Thus, the average follow-up time was 18 months. The effectiveness of the intervention on HbA_1c_ and total cholesterol levels were estimated by the differences between the diabetes populations in the intervention practices and the usual practices with regard to 1) mean HbA_1c_ and total cholesterol, and 2) proportions with HbA_1c_ ≥8%, and proportions with total cholesterol ≥5 mmol/l at follow-up. The differences in mean at follow-up were estimated based on a mixed additive model where adjustments were made for baseline values and random differences between general practices (STATA 12 xtmixed), e.g. analyses were adjusting for clustering within general practices. Furthermore, the differences were adjusted for age (as continuous variable), gender, ethnicity, educational level, cohabitation, and redeemed diabetes medication in the baseline period. The differences in the proportions of patients with a follow-up HbA_1c_ ≥8% and the proportions with a follow-up total cholesterol ≥5 mmol/l were estimated based on mixed additive models that adjusted for baseline status (HbA_1c_ ≥8% and total cholesterol ≥5 mmol/l respectively) and random differences between general practices (SAS 9.3 nlmixed).

The sum scores for the questionnaire scales were calculated where no item-response was missing. Due to a non-normal distributed data, even after log transformation, the sum scores were presented as medians with quartiles. The hypothesis of no difference between medians was tested by a permutations test using general practices as permutation units, 1,000 simulations and the absolute difference in medians as criteria (STATA 12). Confidence intervals for the difference of two medians were found using two-level bootstrapping by resampling general practices and patients within general practice using 1,000 bootstrap samples (STATA 12). Hence, the differences of the medians were also adjusted for clustering.

Subgroup analyses were performed with regard to age, gender and educational level.

Results were presented with 95% confidence intervals (CI) and p values. P values of <0.05 were regarded as statistically significant.

Non-responder analyses were performed for age, gender, educational level, cohabitation and ethnicity by chi_2_-test and by multiple regression analyses.

Regarding the nurse questionnaire data, for each autonomy supportive competence, the number of nurses was counted for the scores from 1–10 (1 = rarely, 10 = always), before and 11 months after the training course. Furthermore, the number of changed autonomy supportive competences per nurse was summarised.

**Figure 2:**
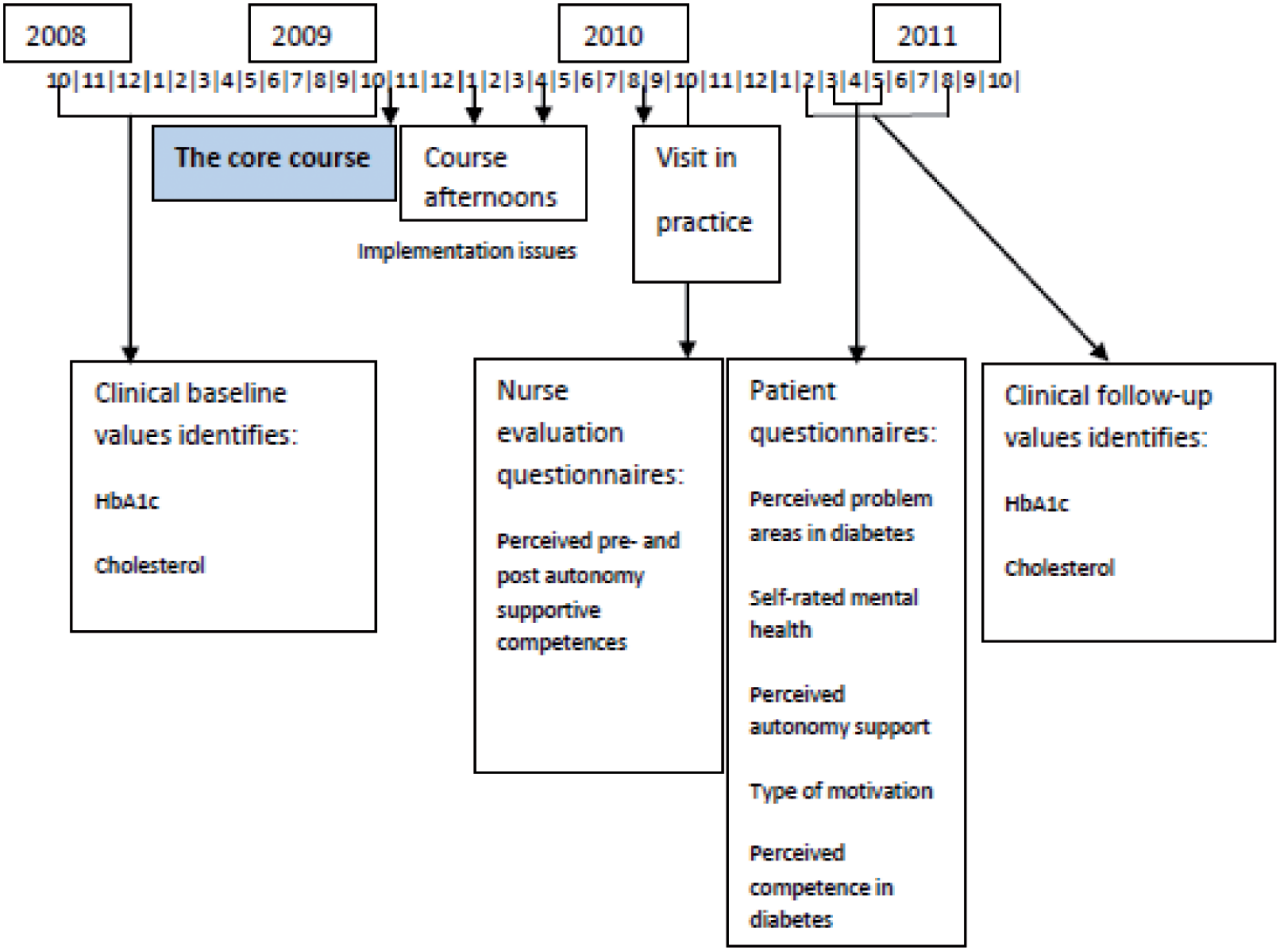
Timeframe of the trial. The trial evaluated the effectiveness of a training course for general practice nurses in applying Self-determination theory in current type 2 diabetes care, DK.

A timeframe of the trial is shown by Figure 2.

### Sample Size and Power

We based the power calculation on the primary outcome HbA1c and a decrease of 0.5% in mean HbA1c [24] in the intervention practices compared to the usual practices. Furthermore, the average number of diabetes patients per practice was assumed to be 50, but we did not expect all to participate in a nurse-led diabetes consultation, i.e. the number of patients per practice was set to 25. From empirical data we estimated the standard deviation to be 0.019. The practices’ ICC were set to 0.01 [25]. Based on this, we needed a total of 30 practices in order to obtain a power of 90% [26].

### Ethical Approval

The Danish Research Ethics Committee concluded that the trial was not to be a biomedical intervention cf The Committee Act no. 402 of the 28 May 2003 §7,1 available at: https://www.retsinformation.dk/Forms/R0710.aspx?id=29142, and because the intervention addressed the nurses, the informed consent of the diabetes population was not required. According to Danish law, approval by the Ethics committee and written informed consent is not required in questionnaire-based and register-based projects. Additional information is available at The National Committee on Health Research Ethics’ webpage in the “Act on Research Ethics Review of Health Research Projects” §14,2. available at: http://www.cvk.sum.dk/English/actonabiomedicalresearch.aspx.

The study was approved by the Danish Data Protection Agency (j.no: 2009-41-3065) and it was registered at ClinicalTrials.gov (Identifier NCT01187069). The data are stored at the Department of Public Health, Aarhus University and at Statistic Denmark.

## Results

### Baseline Characteristics of the Diabetes Population

The baseline characteristics of the diabetes population are shown in Table 1. The mean age was 60.4±8.6 years and 56.5% were men. The median HbA_1c_ at baseline was 6.7% (quartiles: 6.2, 7.6). Among the 1,879 patients where a measurement was performed in the intervention practices, 373 (19.9%) had a baseline HbA_1c_ ≥8% compared with 354 (18.5%) of the 1,910 patients where a measurement was performed in the usual practices. The mean total cholesterol at baseline was 4.6 mmol/l in both groups.

**Table 1:** Baseline characteristics.

Characteristics	Interventionpractices	Usualpractices
Number identified by the algorithm	2,005	2,029
Mean (SD) age (years)	60.2 (8.5)	60.7 (8.6)
Gender, male (%)	1,120 (55.9)	1,159 (57.1)
Living alone (%)	642 (32.0)	653 (32.2)
Immigrant (%)	226 (11.3)	251 (12.4)
Educational-level (%)		
≤10 years	851 (42.4)	858 (42.3)
>10≤15 years	900 (44.9)	922 (45.4)
>15 years	254 (12.7)	249 (12.3)
Diabetes duration (median (quartiles)) years)^a^	8 (4, 14)	8 (4, 15)
HbA_1c_ measured (%)^b^	1,879 (93.7)	1,910 (94.1)
Mean (SD) HbA_1c_ (%)	7.1 (1.3)	7.1 (1.3)
Median HbA_1c_ (%) (quartiles)	6.7 (6.2, 7.7)	6.7 (6.2, 7.6)
Proportion of patients with HbA_1c_ ≥8%	373 (19.9)	354 (18.5)
Total cholesterol measured (%)^b^	1,788 (89.2)	1,821 (89.7)
Mean (SD) total cholesterol (mmol/l)	4.6 (1.0)	4.6 (1.0)
Median total cholesterol (mmol/l) (quartiles)	4.5 (3.9, 5.1)	4.5 (4.0, 5.2)
Proportion of patients with total cholesterol ≥5 mmol/l (%)	532 (29.8)	558 (30.6)
Prescription redemption^b^		
Insulin or oral blood glucose lowering agents	1,516 (75.6)	1,443 (71.1)
only oral blood glucose-lowering-agent	960 (47.9)	887 (43.7)
only insulin	312 (15.6)	307 (15.1)
oral blood glucose-lowering-agent + insulin	244 (12.2)	249 (12.3)
Lipid-lowering-medication	1,488 (74.2)	1,511 (74.5)

a Based on questionnaire responders.

b between 29/10-2008–29/10-2009.

The diabetes population in the intervention practices (n = 20) and in the usual practices (n = 20). Values are numbers unless stated otherwise.

### Effectiveness of the Intervention

#### HbA_1c_

**Figure 3:**
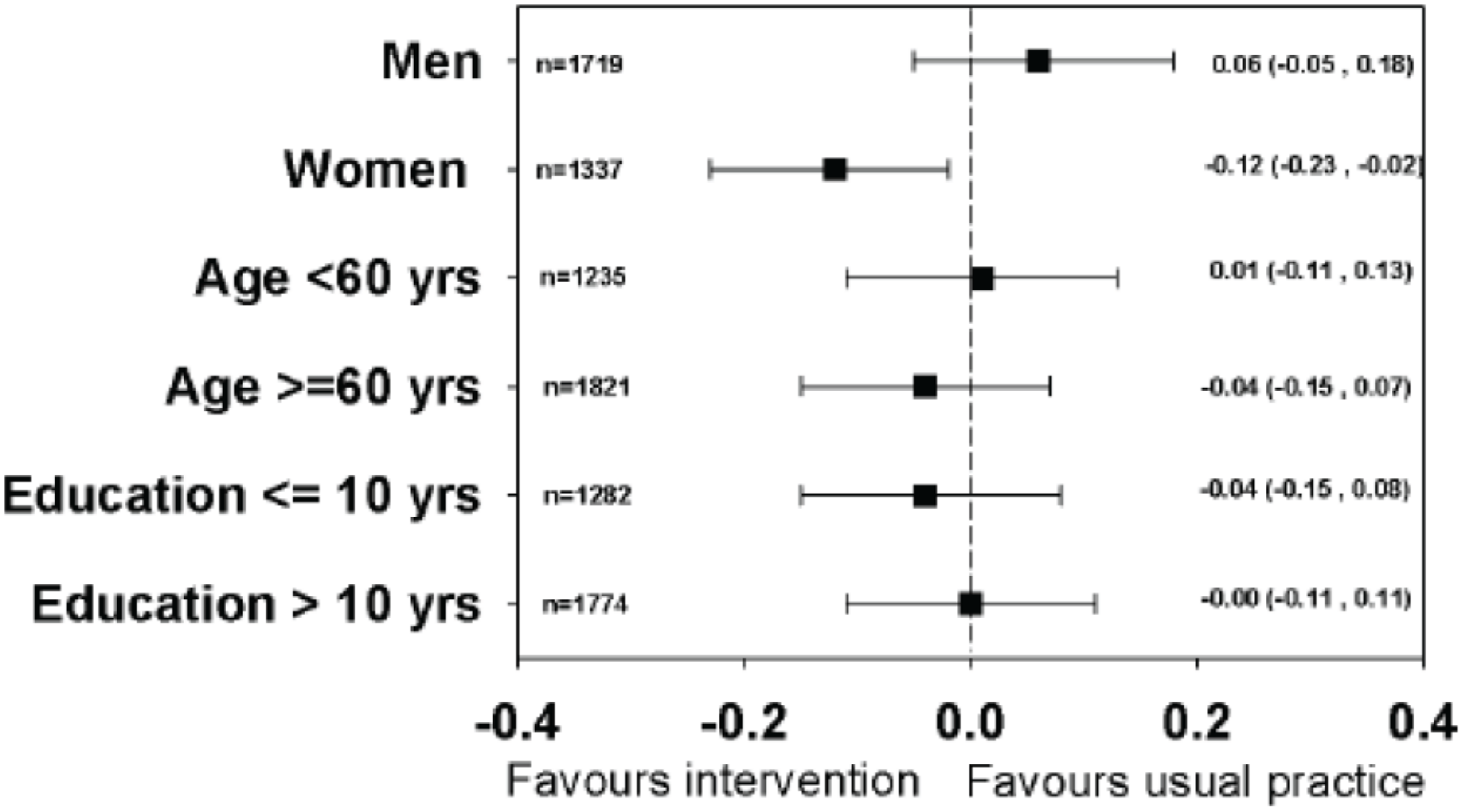
Subgroup analyses. Differences in mean HbA_1c_ adjusted for baseline values and cluster effect in subgroups in intervention practices compared with usual practices 18 months after the core intervention, DK 2011.

The median HbA_1c_ at follow up was 6.8% (quartiles: 6.2, 7.6). No statistically significant difference was found between the groups in the mean follow-up values adjusted for baseline values and clustering (Table 2). Adjustment for age, gender, ethnicity, educational level, cohabitation and diabetes medication redeemed within the baseline period did not change the results. At follow-up, the proportion with HbA_1c_ ≥8% was 18.3% in the intervention practices compared with 18.1% in the usual practices, and, again, no statistically significant difference was found between the groups when figures were adjusted for baseline values and clustering (Table 2). No statistically significant differences were found in the subgroup analyses according to age, educational level and in men, but women in the intervention practices had small, but statistically significantly lower HbA_1c_ levels at follow-up than the women in the usual practices (Figure 3). We observed a −2.3%-points (95% CI: −5.2 to 0.6; p: 0.13) difference in the proportion with HbA_1c_ ≥8% adjusted for baseline and clustering at follow-up in women in intervention practices compared with women in usual practices.

**Table 2:** Intervention effectiveness.

Outcome measure	Eligible no for analysis (%)^a^	Total with data available (%)	Follow up value	Difference atfollow up I-C(95% CI)^b^	P
HbA_1c_ (%)			*Mean (95% CI)*		
Intervention	1,959 (100)	1,513 (77)	7.06 (6.94, 7.17)	−0.02 (−0.11, 0.07)	0.67
Usual	1,987 (100)	1,543 (78)	7.10 (6.98, 7.22)		
HbA_1c_ ≥8% (%)					
Intervention	1,959 (100)	1,513 (77)	18.3 (15.9, 20.7)	−0.6 (−2.7, 1.5)	0.59
Usual	1,987 (100)	1,543 (78)	18.1 (15.7, 20.6)		
Total cholesterol (mmol/l)					
Intervention	1,959 (100)	1,147 (59)	4.4 (4.3, 4.4)	0.08 (0.01, 0.15)	0.02
Usual	1,987 (100)	1,048 (53)	4.3 (4.3, 4.4)		
Total cholesterol ≥5 mmol/l (%)					
Intervention	1,959 (100)	1,147 (59)	23.4 (21.9, 29.1)	2.6 (−0.2, 5.4)	0.07
Usual	1,987 (100)	1,048 (53)	22.1 (19.8, 24.6)		
PAID			*Median (quartiles)*		
Intervention	1,112 (100)	1,019 (92)	13.8 (3.8, 28.8)	1.25 (−1.71, 4.21)	0.31
Usual	1058 (100)	951 (90)	12.5 (3.8, 26.3)		
SF12, mcs					
Intervention	1,112 (100)	960 (86)	52.2 (43.0, 57.8)	0.99 (−1.02, 3.00)	0.15
Usual	1,058 (100)	907 (86)	51.2 (42.4, 57.3)		
HCCQ					
Intervention	768 (69)^c^	697 (91)	6.2 (5.0, 7.0)	0.17 (−0.18, 0.51)	0.43
Usual	732 (69)^c^	670 (92)	6.0 (5.0, 7.0)		
TSRQ-medication					
Controlled motivation					
Intervention	948 (85)^d^	858 (91)	4.8 (3.8, 5.8)	0.00 (−0.39, 0.39)	1.00
Usual	882 (83)^d^	798 (91)	4.8 (3.8, 6.0)		
Autonomous motivation					
Intervention	948 (85)^d^	868 (92)	6.0 (5.0, 7.0)	0.00 (−0.20, 0.20)	1.00
Usual	882 (83)^d^	805 (91)	6.0 (5.0, 7.0)		
TSRQ-diet and physical activity					
Controlled motivation					
Intervention	991 (89)^e^	882 (89)	4.3 (3.2, 5.5)	−0.08 (−0.43, 0.26)	0.70
Usual	936 (88)^e^	813 (87)	4.3 (3.5, 5.5)		
Autonomous motivation					
Intervention	991 (89)^e^	891 (90)	6.6 (5.8, 7.0)	0.00 (−0.24, 0.24)	0.98
Usual	936 (88)^e^	836 (89)	6.6 (5.8, 7.0)		
PCDS					
Intervention	1,112 (100)	1,009 (91)	6.5 (5.8, 7.0)	0.00 (−0.34, 0.34)	0.97
Usual	1,058 (100)	966 (91)	6.5 (5.8, 7.0)		

a Blood test analyses: people identified in the diabetes register at baseline and alive at follow up and for the questionnaire scale analyses: furthermore, responding to the questionnaire and not reported having no diabetes.

b Adjusted for cluster effect and for the blood test analyses; also for baseline values.

c Stated participation in a diabetes consultation provided by a practice nurse during the past 12 months.

d Stated taking diabetes medication.

e Stated following advices on diet and physical activity.

Differences at 18 months of follow up between the diabetes population in 20 usual practices and in 20 intervention practices. The intervention practices had been offered a training course for general practice nurses in applying the Self-determination Theory in current type 2 diabetes care, DK 2011.

#### Total cholesterol

At follow-up, the mean total cholesterol was 4.4 mmol/l in the intervention practices compared with 4.3 mmol/l in the usual practices. The 0.08 mmol/l (95% CI: 0.01 to 0.15) difference was statistically significant. The proportion with a follow-up cholesterol measurement was 6%-points (95% CI: 3 to 9) higher in the intervention practices compared with the usual practices. This tendency was also present in the subgroup analyses regarding the men, the diabetes population <60 years old, and the diabetes population with >10 years education. Adjustment for age, gender, ethnicity, educational level, cohabitation and redeemed diabetes medication in the baseline period did not change the results. The difference in the proportion of the diabetes population with total cholesterol ≥5 mmol/l was not statistically significant between the groups as such (Table 2), or in the subgroup analyses.

#### Well-being

The overall median score for PAID at follow-up was 12.5 (quartiles: 3.8, 27.5). No statistically significant difference was found between the groups (Table 2). The overall median score of mcs, SF12 was 51.8 (quartiles: 42.8, 57.8) and, again, no statistically significant difference was found between the groups. No statistically significant differences were found in the subgroup analyses according to age, educational level or in men, but women in the intervention practices had a median score of mcs, SF12 of 51.0 (quartiles: 42.6, 57.3) compared with 48.4 (quartiles: 39.4, 57.1) in women in the usual practices, and the difference at 2.6 score-points (95% CI: −0.8 to 5.9) adjusted for clustering had a p-value: 0.01.

#### Motivation

No statistically significant differences between the groups were found in the scores for the SDT scales (Table 2). No other statistically significant differences were found in the subgroup analyses according to age, gender and educational level.

### Intervention Delivery

**Figure 4:**
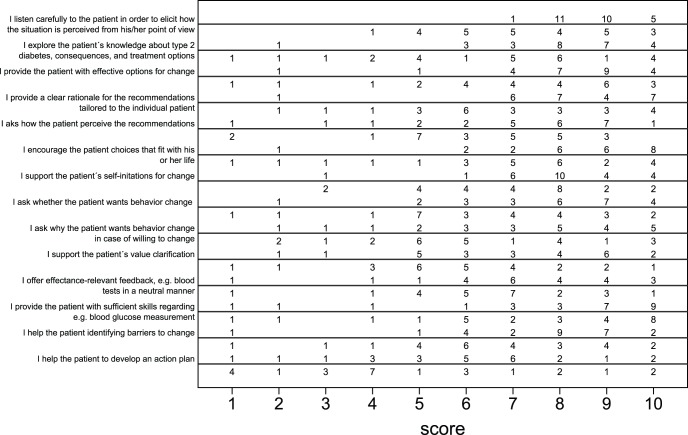
Evaluation of the training course on the nurses’ self-perceived autonomy supportive competences. The number of nurses distributed on their scores from 1–10 (1 = rarely, 10 = always) *before* (below the line) and *11 months after* (above the line) the training course for each autonomy supportive competence, DK 2011.

A total of 34 nurses from 19 of 20 intervention practices had received the core intervention, and 22 nurses from 13 practices had completed the full course (Figure 1). Twenty-seven nurses from 18 of the 20 intervention practices evaluated the course. Figure 4 shows for each autonomy supportive competence, the number of nurses distributed on their scores from 1–10 (1 = rarely, 10 = always) before (below the line) and 11 months after (above the line) the training course. It shows that the nurses perceived that they improved the autonomy supportive competences. For example, regarding the question “I listen carefully to the patient in order to elicit how the situation is perceived from his/her point of view”, 12 nurses scored their performance to be 8 or more before the course, whereas 26 nurses scored their performance to be 8 or more after the course.

One nurse (4%) did not answer the questions, and 7/27 (26%) reported no perceived changes in the autonomy supportive competences. The remaining 19/27 nurses (70%) reported perceived improvement in at least three of the autonomy supportive competences. We do not know whether the nurses who did not complete the evaluation questionnaire have perceived changes in the autonomy supportive competences. The worst case scenario is that 19/34 (56%) perceived improvement in some of the autonomy supportive competences, and the best case scenario is that 27/34 (79%) perceived improvement in some of the autonomy supportive competences. Overall, the nurses reported that they had achieved most from the course in terms of improved communication skills. A total of 24 of the 27 (89%) nurses reported that they had distributed worksheets to patients, but only 1 of 27 (4%) reported that she had distributed at least one patient work sheet to all the diabetes patients attending her consultations.

A total of 69% of the diabetes population who completed the questionnaire, reported that they had participated in a nurse-led type 2 diabetes consultation during the follow-up period (Table 2). The median score on the patients’ perceived autonomy support (HCCQ) was higher in the intervention practices compared to the usual practices, but the difference was not statistically significant (Table 2).

### Characteristics of Patients with Missing Data

Non-response to the questionnaire was statistically significantly associated with the following characteristics; younger age, being an immigrant, having ≤10 years of education, living alone and baseline HbA_1c_ ≥8%. Even when these characteristics were adjusted for each other, they all remained statistically significant.

Younger age was statistically significantly associated with not having a follow-up and a baseline HbA_1c_. Younger age and having a baseline HbA_1c_ ≥8% were found to be independently associated with drop-out in the cholesterol analyses. No social determinants for drop out were found in the blood test analyses.

The proportion with no follow-up cholesterol measurement was higher in the usual practices than in the intervention practices, and the total cholesterol baseline values were higher among the patients with no follow-up cholesterol value (mean 4.7 mmol/l) compared to the patients with a follow-up cholesterol value (intervention: mean 4.5 mmol/l; usual: mean 4.6 mmol/l).

## Discussion

### Main Findings and Comparison with Existing Literature

This trial demonstrated that offering a training course for GP nurses in applying SDT in current type 2 diabetes care had no noticeable effect compared with usual practice measured by HbA_1c_ and total cholesterol levels and the well-being at 18 months of follow-up in a comprehensive register-based diabetes population. The difference in mean total cholesterol between the randomisation groups was 0.08 mmol/l (95% CI: 0.01 to 0.15) at follow-up, which favoured usual practice. However, the mean total cholesterol values were below the recommended threshold (4.5 mmol/l) in both groups at follow-up. Furthermore, a higher proportion of the diabetes population had a follow-up total cholesterol measurement in the intervention practices compared with the usual practices, and the patients without a follow-up measurement had higher levels of baseline total cholesterol than those with a follow-up measurement.

The predefined subgroup analyses showed small, but statistically significant differences in mean HbA_1c_ and self-reported mental health in favour of the intervention among the women. Power calculation was not performed regarding the subgroup analyses. However, the precision of the results are shown by 95% CIs. Regarding the difference in the median scores of mcs, SF12, the 95% CI indicated that the difference could be <0.The discrepancy between the significance testing and the confidence interval for the observed difference can appear when using different methods with different assumptions.

The evaluation of the educational process showed that 70% of the nurses who completed the questionnaire perceived that their autonomy supportive skills had improved, especially their communication skills. The process evaluation also showed that implementation of the patient work- sheets was incomplete.

The method Motivational interviewing (MI) has been assumed to be largely in accordance with SDT [27], and comparisons with MI interventions in diabetes care are performed in the following. Heinrich E et al. conducted a highly pragmatic trial similar to ours aiming to embed improved communication skills into daily general practice through a MI-based training course for GP nurses [28]. This intervention showed improved effects on locus of control and knowledge in volunteers with type 2 diabetes, but it also showed adverse effects on fat intake and HDL-cholesterol. No effects were found on multiple outcome measures including HbA_1c_, total cholesterol and quality of life. Henrich et al. found that their results could partly be explained by a too low training intensity of the nurses and limited time for interaction with patients within the framework of existing diabetes care.

Explanatory trials evaluating SDT-based interventions in diabetes care have shown varying results. Recently, a one-year MI programme was evaluated in a Danish diabetes clinic setting including volunteers with type 1 and type 2 diabetes [29]. Although the study did accomplish a rise in health care providers’ MI competences and a high degree of intervention delivery, the study showed no 12- or 24-month effect on PAID or HbA_1c_. A high level of usual care including a patient education programme based on Guided Self-Determination may explain the absence of differences between the randomisation groups. The approach in Guided Self-Determination is consistent with SDT and has as previously mentioned, shown an effect in diabetes care [10].

None of the above-mentioned studies have investigated the gender differential effect of the interventions. West, et al. investigated the effect of adding five individual MI sessions to a group-based behavioural obesity treatment programme for overweight women with type 2 diabetes and reported an effect on weight loss and a decline in HbA_1c_ [30].

### Strengths and Limitations of the Study

We evaluated the effect of a training course with multiple components as a whole under the conditions in which it would be applied. A quality of the present trial is that the basis of the intervention components and all activities are well-described [11].

The near-complete coverage of Danish registers allowed us to evaluate the effectiveness in a comprehensive diabetes population. A limitation of the registers is the inability to distinguish between types of diabetes. However, the number of patients with type 2 diabetes far exceeds the number of patients with type 1 diabetes (85%∶15%), and there should be no reason to assume an unequal distribution between the intervention and the usual practice group. The inclusion of the type 1 diabetes population may however, have contributed to a dilution of a potential effect of the intervention because type 1 diabetes care is primarily delivered by out-patient clinics. For example, the found differences of −0.12%-points in HbA1c and of 2.6 score-points on the 0-100-SF12, mcs scale in our female population could be biased towards the null-hypothesis due to dilution because of the inclusion of the very comprehensive diabetes population.

Our recruitment strategy of the general practices was a realistic scenario for future training courses. A previous study showed that the eligible practices for the present trial were associated with a higher quality of diabetes management when compared with practices with no nurse(s) employed and also when compared with practices that did not respond to that survey [5]. This indicates less room for improvement and a higher level of engagement in diabetes management in the included practices. The lack of knowledge about the activities performed in usual practice is a limitation of our study. The allocation to the usual practice group may have tempted the nurses in the usual practices to join courses similar to the one offered in the intervention practices. On the other hand, each contact to the usual practices could have enhanced focus on communication and provoked that they stopped being “usual” [31].

We used different types of patient outcomes to measure different stages in the expected process from motivation support to improved well-being and changed clinical outcomes. This could speak for multiplicity correction, but because different models and methods were required for the analyses of the outcome measures, a test of no effect based on correlated outcomes was infeasible.

Well-being is directly relevant for the patients, but the questionnaire scales’ sensitivity to detect differences caused by interventions is not clear. The ceiling effects in the motivational outcome measures; the SDT scores and the low levels of the PAID-scores showed very little room for improvement.

Avoiding complications is highly relevant for the patients and the HbA_1c_ - and cholesterol levels are valid surrogates for the development of complications [4]. It is also evident that health behavioural treatment such as physical activity, diet and medication adherence impacts HbA_1c_ and cholesterol values [4], but we had no valid information on these aspects for this study. Hence, we decided to use HbA_1c_ and total cholesterol measurements as indicators of some occurrence of the guideline recommendations. However, the duration of the follow-up may have been too short to detect a difference in these outcomes. This in particular, because we evaluated the effect under real-life conditions and therefore e.g. made no attempt to ensure the patients’ participation in a diabetes consultation provided by the nurse at certain times during the follow-up time [32], [33]. A total of 69% of the questionnaire responders reported they had attended a nurse-led diabetes consultation during the follow-up time.

Whether the current framework of approximately 15 minutes quarterly consultations is adequate for the nurses to sufficiently stimulate the patients’ process of being autonomously motivated could be debatable. A Swedish intervention included besides 1) training of primary care nurses to put more emphasis on the patients’ understanding of their disease in the current framework of diabetes care, also 2) a comprehensive nine months programme with 20 hours of reflective group-discussions on living with diabetes. This explanatory trial showed an intervention effect in terms of statistically significant lower HbA1c-levels in the intervention group compared with the control group after one year (−0.94%-points) and after five years (−1.34%-points) [34], [35].

The present trial was not designed to evaluate whether the training course would work under ideal circumstances. As expected under normal, real-life conditions, not all the intervention practices participated in all or even in parts of the course (Figure 1). This kind of “non-compliance” is included in the real-life effect [36]. Obtaining data on implementation issues could be an intervention component that induces an effect in itself and will not be included in the future intervention [37]. Therefore, we did not e.g. observe whether the training course succeeded in improving the nurses’ autonomy supportive competences in the diabetes consultations. However, the participating nurses completed an evaluation questionnaire, which we assumed would be realistic for the future. A total of 70% of the nurses who completed the evaluation questionnaire, reported perceived improvement in at least three of the autonomy supportive competences. This kind of information is potentially biased, but it does however give a picture of the nurses’ perceived benefits.

The underlying assumptions behind sample size calculation of a cluster randomised pragmatic trial like ours can be discussed, e.g. the ICC and the effect size in use [38]. Likewise, other methods could have been used for the sample size calculation [39]. It should be noticed, that we enrolled more general practices and patients than suggested by our power calculation; there was a lot of interest among the practices for participating in the study and it turned out that there was much more patients with diabetes associated with the practices than expected. We report all estimated effect with confidence intervals adjusted for the clustering of the data. The confidence intervals on the primary outcome HbA_1c_ and total cholesterol are narrow and they all show that if effectiveness of the intervention exists, then it is small. However, it could be argued that even small effect sizes found at the diabetes population level in this pragmatic trial design could be worthwhile because of the low intervention costs. The costs of this intervention solely comprised the costs of the education of the nurses because the intervention was delivered to the patients in the framework of existing diabetes care and thereby without additional expenditure. The magnitude of relevant effect sizes seen in relation to intervention costs deserves more attention in the planning of future interventions evaluated in pragmatic trial designs.

## Conclusions

Offering a training course for GP nurses in applying SDT in current type 2 diabetes care had no effect compared with usual practice measured by HbA_1c_ and total cholesterol levels and well-being at an 18 month follow-up in a comprehensive register-based diabetes population. Subgroup analyses suggested a possible effect in women on HbA_1c_ and well-being, which deserves further attention.

## Supporting Information

Checklist S1 CONSORT 2010 checklist of information to include when reporting a cluster randomised trial.
(DOCX)

Checklist S2 CONSORT Checklist of Items for Reporting Trials of Nonpharmacologic Treatments.
(DOC)

Protocol S1 Trial protocol detailed description.
(PDF)

Protocol S2 The first draft.
(DOC)
