# Supplementary material for: Effectiveness of a Training Course for General Practice Nurses in Motivation Support in Type 2 Diabetes Care: A Cluster-Randomised Trial
Source: PLoS One. 2014 May 5;9(5):e96683. doi: 10.1371/journal.pone.0096683 (PMC4010512; doi:10.1371/journal.pone.0096683)
Supplement: Protocol S1 — Trial protocol detailed description. (PDF) [file pone.0096683.s003.pdf]

Juul et al. BMC Family Practice 2011, 12:130
http://www.biomedcentral.com/1471-2296/12/130

BMC Family Practice

STUDY PROTOCOL Open Access

A cluster randomised pragmatic trial applying Self-determination theory to type 2 diabetes care in general practice

Lise Juul[1*], Helle T Maindal[1], Vibeke Zoffmann[2], Morten Frydenberg[3] and Annelli Sandbaek[1]

Abstract

Background: Treatment recommendations for prevention of type 2 diabetes complications often require radical and life-long health behaviour changes. Observational studies based on Self-determination theory (SDT) propose substantial factors for the maintenance of behaviour changes and concomitant well-being, but experimental research is needed to develop and evaluate SDT-based interventions. The aims of this paper were to describe

1) the design of a trial assessing the effectiveness of a training course for practice-nurses in autonomy support on patient-perceived motivation, HbA1, cholesterol, and well-being among a diabetes population,
2) the actual intervention to a level of detail that allows its replication, and
3) the connection between SDT recommendations for health care-provider behaviour and the content of the training course.

Methods/Design: The study is a cluster-randomised pragmatic trial including 40 Danish general practices with nurse-led diabetes consultations, and the associated diabetes population. The diabetes population was identified by registers (n = 4034).
The intervention was a 16-hour course with interactive training for practice nurses. The course was delivered over 4 afternoons at Aarhus University and one 1/2 hour visit to the practice by one of the course-teachers over a period of 10 months (0, 2, 5, 10 mths.). The intervention is depicted by a PaT Plot showing the timeline and the characteristics of the intervention components.
Effectiveness of the intervention will be assessed on the diabetes populations with regard to well-being (PAID, SF-12), HbA1c- and cholesterol-levels, perceived autonomy support (HCCQ), type of motivation (TSRQ), and perceived competence for diabetes care (PCD) 15-21 months after the core course; the completion of the second course afternoon. Data will be retrieved from registers and by questionnaires.

Discussion: Challenges and advantages of the pragmatic design are discussed. In a real-world setting, this study will determine the impact on motivation, HbA1c, cholesterol, and well-being for people with diabetes by offering a training course in autonomy support to practice-nurses from general practices with nurse-led consultations.

Trial registration: ClinicalTrials.gov: NCT01187069

* Correspondence: lise.juul@alm.au.dk
[1]Department of Public Health, Section for General Practice, Aarhus University, Denmark
Full list of author information is available at the end of the article

BioMed Central

© 2011 Juul et al; licensee BioMed Central Ltd. This is an Open Access article distributed under the terms of the Creative Commons Attribution License (http://creativecommons.org/licenses/by/2.0), which permits unrestricted use, distribution, and reproduction in any medium, provided the original work is properly cited.

Background

A great body of evidence exists on treatment recommendations for preventing type 2 diabetes complications [1-3]. These recommendations often require radical and life-long health behaviour changes for people with type 2 diabetes. Self-determination theory (SDT) [4], a psychological theory on motivation, describes the importance of the quality of the motivation for making behaviour changes. The quality of motivation depends on whether the regulation of actions are imposed by others or oneself (controlled motivation) or by the true feeling of free choice and personal significance of the outcomes attained by the actions (autonomous motivation). In observational studies, autonomous motivation has been found to be associated with improved quality of life, improved medication adherence, less depression, better diet, and improved cholesterol- and A1C-levels among people with type 2 diabetes [5-8]. Furthermore, observational studies based on SDT propose that support from health care providers can influence the quality of motivation for behaviour changes among people with type 2 diabetes. In Denmark, type 2 diabetes care primarily is delivered in general practice. In Danish general practices, nurses provide an increasing number of tasks including diabetes consultations [9]. Accordingly, it is important to further develop the content of nurse-led diabetes consultations, and more research is needed to tailor interventions aiming to improve autonomy support for people with type 2 diabetes in general practice.

The aims of this paper were to describe

1. the design of a trial assessing the effectiveness of a training course for practice-nurses in autonomy support on patient-perceived motivation, HbA1, cholesterol, and well-being among a diabetes population,
2. the actual intervention to a level of detail that allows its replication, and
3. the connection between SDT recommendations for health care-provider behaviour and the content of the training course.

Methods/Design

Study design

The study was conducted as a cluster-randomised controlled trial highly pragmatic in attitude including 40 Danish general practices and their diabetes populations.

The context

Danish general practice

In Denmark, general practice is the primary access to the health care system, and 98% of the Danish population are registered with a selected general practice. Danish general practices are independent contractors within the public health service; the regional health authorities and they are remunerated on a combination of fee-for-service and capitation basis (75/25). The publicly funded (pre-paid by taxes) health care system ensures all citizens free general practice service [10].

Practice nurses

Practice nurses are employed by the general practitioners. The nurses provide a variety of tasks depending on their competences and the organisation of the clinic. Common tasks include weight control, vaccination of adults, blood pressure checks, lung function tests, alcohol abuse treatment and dietary counseling [9]. Practice nurses are qualified registered nurses. Their training in Denmark lasts 31/2 year and consists of both a theoretical and a clinical part. After qualifying, Danish nurses have several opportunities for post graduate education. However, no diploma education for practice nurses was available at the beginning of the training course "Practice nurses and Type 2 diabetes".

Recruitment

Practices

In August 2009, a questionnaire about nurses and type 2 diabetes-consultations and an invitation to participate in the present study were sent to 258 general practices (registration numbers) from the former county of Aarhus in Denmark (Figure 1). A total of 50 practice registration numbers corresponding to 40 different addresses were included in this study based on the following inclusion criteria:

1) that more than 50% of the people with type 2 diabetes, who had participated in a diabetes-consultation during the past year from the project invitation-date, had attended a consultation with a nurse at least once (confirmed by the practice). A diabetes-consultation was defined as a consultation including examinations, such as blood tests and blood pressure measurement as well as a conversation about living with type 2 diabetes or treatment modalities, according to the three-month consultations in the National guidelines regarding type 2 diabetes care [11],

2) enrolment to the project before registration deadline (three weeks following the date of invitation).

To avoid contamination of the intervention to control-practices, practice registration numbers with the same addresses were defined as one practice. Thus, 40 practices were randomised into two groups, and allocated in a way that the distributions of associated patients were approximately the same in the two groups. Randomisation was performed by a statistician who was blinded to the identity of the practices.

The diabetes population

The diabetes population associated with the included practices in January 2011 was retrieved from the Danish Regional Chronic Database where people with diabetes

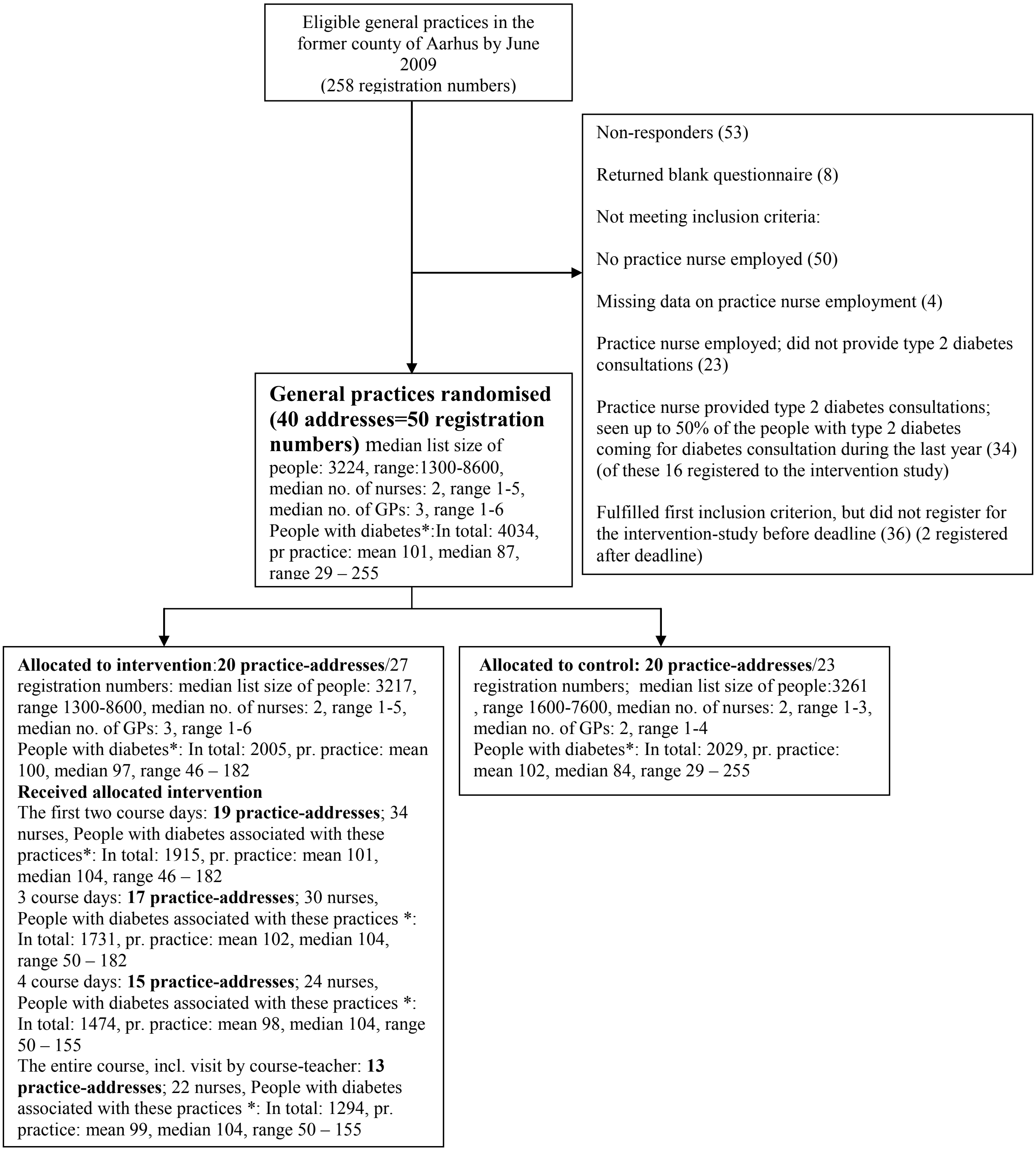

Figure 1 Flow diagram of inclusion, randomisation and allocated intervention received among 258 general practices assessed for eligibility, the former county of Aarhus, DK, 2009. * People identified by an algorithm based on health registers 2008-2009, and associated with the practices in January 2011.

are identified by an algorithm based on health registers [12]. The health registers consisted of The National Patient Registry, The National Health Insurance Service Registry, the prescription database, and the laboratory databases in the region. People at the ages of 40-74 years in October 2009 who fulfilled one or more of the following criteria: 1) at least one redeemed prescription on diabetes-medication (ACT-code: A10A (insulin) or/ and A10B (oral blood-glucose-lowering agents) during October 2008 - October 2009, 2) at least three HbA1c

measurements during October 2008 - October 2009, and/or 3) at least one HbA1c ≥6,4% between April 2008-October 2009, were included in the study population (n = 4034).

The intervention

The intervention was a training course for practice nurses delivered between October 2009 - September 2010.

The intervention was depicted by a PaT Plot, proposed by Perera et al, showing the timeline and the characteristics (fixed or flexible) of the intervention components [13] (Figure 2 and 3).

Underlying theory

The intervention was based on Self-determination theory (SDT), which describes different kinds of motivation and facilitators of improved and maintained motivation [4] (Figure 4). SDT distinguishes between autonomous and controlled motivation. The motivation is controlled, when actions are externally regulated or regulated by introjections, and the motivation is autonomous, when actions are regulated by identification or integrated. The process of being autonomously motivated to perform an activity in order to attain some separable outcome is called internalisation. According to Ryan and Deci, internalisation and integration can be facilitated by social conditions supporting basic psychological needs for autonomy, relatedness, and competence [4]. In other words, the feeling of choice and volition with respect to one's own goal or behaviour, the feeling of interest and support of others, and the feeling that one can accomplish the behaviours and reach the goal, are crucial for motivation, the persistence of the motivation, and well-being; And, satisfying these needs can be supported by important others, e.g. health care providers.

The connection between SDT-based recommendations on health care provider behaviour and the course content

Concrete recommendations on health care provider behaviour based on SDT have been suggested [14,15], and the training course for practice nurses described below was conducted to meet these recommendations.

Structure and process

The course was arranged as four-hour sessions over four afternoons. The course was initiated with two coherent afternoons comprising the core intervention, followed by two single afternoons with an implementation purpose, two and five months later. The practice nurses were paid their normal wages for participating in the course. Ten months after course-initiation, the practice nurses were given the opportunity to be visited in the practice by one of the course-teachers.

The teaching on the course was primarily performed by two nurses with several years experience of consultations for people with diabetes, autonomy support, and learning processes. The lesson about current treatment recommendations of people with type 2 diabetes was provided by a general practitioner, who was also an Associate Professor in general practice, and researcher in the field of type 2 diabetes.

The ratio between the presentations and interactive learning [16] was 1:3. The interactive learning was conducted in group exercises, work-shops, and plenum discussions, all supervised by the teachers. The teaching was intended to be autonomy-supportive, based on the evidence from the association between autonomy-supportive teaching climates and behavioural changes in the participants [17].

Content

The course content was 1) Patient - health care provider relationships, 2) Communication-skills, 3) Patient-worksheets, 4) Current treatment recommendations of type 2 diabetes, and 5) Implementation of the course content in daily practice. The content according to 1), 2), and 3) was based on a course in another trial, Guided Self-Determination, performed in a Danish outpatient diabetes clinic [18]. This intervention showed an effect on perceived autonomy support and improved HbA1c-values among people with type 1 diabetes [18].

Patient - health care provider relationships

In a qualitative study design, Zoffmann et al. identified different patient - health care provider relationships with different potential for change among people with diabetes [19]. These relationships: I-you distant provider dominance, I-you blurred sympathy, and I-you sorted mutuality were presented at the first course afternoon. Furthermore, they were included in communication exercises on day 2, 3, and 4. Predominantly meeting the people with I-you distant provider dominance seems to be similar to what Sheldon, et al. define as controlling health care providers [14], and predominantly meeting the people with I-you sorted mutuality seems to be in accordance with autonomy support, if the health care provider is conscious of being neutral and non-judging. The focus of the communication in the I-you sorted mutuality relationships was presented as an empathic approach where the patient's perspectives and values were elicited, acknowledged, and explored.

Communication-skills

The communication skills: mirroring, active listening, and values clarification response [18] were presented on the second course afternoon, and were used in different exercises on day 2, 3, and 4.

Mirroring was defined as telling the other person; what you observe, followed by a pause. For an example, repeating the person's last few words in order to make him feel heard, and giving him the opportunity to hear himself. The pause encourages reflection, or perhaps more elaboration [20].

**Timeline**	**Intervention**<br><br>**(A training course in autonomy support for practice nurses)**	**Control**<br><br>**(Usual practice)**
2.5 months before course-start	a	
Randomisation		
2 months before course-start	b c	b
14 days before course-start	d	
The core course	1 e f	
The day after participation in the core course	Follow-up time begins	
1 month	g	
2.5 months	h	
3 months	2	
4 months	i	
5 months	h	
5.5 months	3	
9-10 months	4	
9-10 months	j	
11 months	k	
15-21 months	Follow up time ends	

Explanations:

a	Questionnaire concerning nurses and type 2 diabetes-consultations and invitation to participate in the study

Figure 2 Graphical depiction of the timeline and the content of the "Practice nurses and Type 2 diabetes"-intervention. Squares reflect the fixed elements, e.g. printed materials. Circles reflect the activities that are flexible, e.g. interaction between participants. This graphical method was proposed by Perera et al [13],(part 1 - to be continued in Figure 3).

Active listening was defined as telling the other person how you have understood the message in his total communication. The recipient tries to understand the feelings of the sender, and the meaning of the message. Afterwards, without valuing, analysing, or giving advices, the recipient formulates her perception of the message, and sends it back to the sender in order to make him validate the perception [21].

b	Letter about allocation to intervention- or control group
c	Course-programme enclosed letter b
d	"Looking forward to see you"-letter and participation-list
(1)	Course on autonomy-support for a group of 17 nurses; two successive afternoons of 4 hours duration. The course was held for 2 groups of nurses within 2 weeks. Educational format: presentations, group work.
e	**Power-point presentations, all handed out:**<br>• Self-determination theory (20 minutes)<br>• Types of relationships (I-you distant provider dominance, I-you blurred sympathy, I-you sorted mutuality) (40 minutes)<br>• The meaning of time in relation to autonomy support (15 minutes)<br>• Introduction to patient-work-sheets as tools to support autonomous motivation (1."What do you currently find difficult about living with your type 2 diabetes?", 2."Your plans to change your way of life", 3."Action-plan")(40 minutes)<br>• How to use this method in daily practice? (20 minutes)<br>• Current treatment recommendations for type 2 diabetes (45 minutes)<br>• Communication skills (Mirroring, Active listening, Values clarification response) (45 minutes)<br><br>**Group-exercises (printed material handed out) (3*45 minutes):**<br><br>1) exemplifying mirroring, active listening and value clarifying response to defined cases and statements<br><br>2) communication-exercises using nurse-work-sheets (1."Challenges of collaboration with people with type 2 diabetes", 2."Your plans to change your approach to people with type 2 diabetes", 3. "Action-plan") **handed out the day before**
f	Three articles: "Educating to life with diabetes", "Communication skills", and "The relation between patient and professional" by Vibeke Zoffmann were handed out
g	Letter with information on next course-day. Encouraged to video-tape or describe a consultation with a person with type 2 diabetes. Encouraged to propose suggestions for next course programme
h	"Looking forward to see you"-letter; programme, participation-list, and nurse-work-sheets
(2)	Course, implementation 1: for a group of 14-16 nurses on 1) experiences with implementing autonomy-support, and 2) communication training; one afternoon of the duration of 4 hours. The course was held for 2 groups of nurses within 2 weeks. Educational format: group work, plenum discussions
i	Letter with information on following course-day. Encouraged to propose suggestions for next course programme
(3)	Course, implementation 2: for a group of 11-13 nurses on 1) experiences with implementing autonomy-support, 2) relevant information for people with type 2 diabetes, and 3) communication training; one afternoon of 4 hours duration. The course was held for 2 groups of nurses within 2 weeks. Educational format: workshops, plenum discussions
(4)	½ hour visit to the nurse in the practice by one of the course teachers with the agenda: 1) Expectations for the meeting, 2) What do you currently find difficult about collaborating with people with type 2 diabetes?, and 3) Do you have any specific needs of knowledge about collaborating with people with type 2 diabetes?
j	A description on "Self-determination theory and application in clinical practice" was handed out to the nurses, based on [14]
k	An evaluation-questionnaire on the course was sent to all the nurses, who partly or fully completed the course

Figure 3 Graphical depiction of the timeline and the content of the "Practice nurses and Type 2 diabetes"-intervention. Squares reflect the fixed elements, e.g. printed materials. Circles reflect the activities that are flexible, e.g. interaction between participants. This graphical method was proposed by Perera et al [13], (part 2 - Figure 2 continued).

Mirroring is in accordance with simple reflection from motivational interviewing, while active listening is in accordance with summary reflection from the same [14].

The purpose of value clarification is to initiate a process to reconsider and clarify own values, and value clarification response (VCR) is one way to promote this process. The purpose of VCR is to give the patient

Type of motivation	Amotivation	Extrincic motivation				Intrinsic motivation
Regulatory styles	**Non-regulation**	**External regulation**	**Introjected regulation**	**Identified regulation**	**Integrated regulation**	**Intrinsic regulation**
Regulatory processes	Nonintentional, Nonvaluing, Incompetence, Lack of control.	Compliance, External rewards and punishments.	Self-control, Ego-Involvement, Internal rewards and punishments.	Personal importance, Conscious valuing	Congruence, Awareness, Synthesis with self.	Interest, Enjoyment, Inherent satisfaction.
		**Controlled motivation**		**Autonomous motivation**		

Figure 4 The Self-Determination Continuum showing types of motivation with their regulatory styles and processes, adapted from [4].

something to think about concerning the way he acts as well as his attitudes, interests, and assessments. VCR was defined as a reply or a question stimulating reflection and self-insight, and it can often not be answered right away, but has a potential to stimulate a development process [22]. Examples of VCR could be "What does taking preventive medication mean to you?", or "What is important in your life?". Value clarification is also recommended by Sheldon, et al. [14].

Patient-worksheets

Three patient-worksheets were introduced with the purpose of stimulating the person with type 2 diabetes's reflection process between the consultations. We stressed that the person should be invited to use worksheets, but that it was entirely their own decision whether they wanted to use them or not. Two of the worksheets were from the method Guided self-determination (GSD) [18], and the action plan was from the Chronic care model (presented in [23]).

One was a reflection sheet with two explorative questions "What do you think has worked, or is working in relation to your life with type 2 diabetes?" and "What do you currently find difficult about living with type 2 diabetes?". The purpose of this work-sheet was to elicit and acknowledge the person's perspective, and to initiate the person's involvement in problem solving. Another worksheet from GSD, "Your plans to change your way of life" was inspired by the Stages of change model developed by Prochaska and Diclemente. It contained a number of statements about behaviours with importance for type 2 diabetes. The person with type 2 diabetes was encouraged 1) to assess what was currently difficult or challenging in his life, and 2) to assess whether he was interested in changing his behaviour or not. This worksheet was intended to give an overview of the person's stages of changes in relation to different recommendations of living with type 2 diabetes, and to acknowledge the option of no willingness to change. Sheldon et al. wrote that people willing to change a behaviour within a 2-4 week period could be thought of as being motivated for making an attempt [14]. The action-plans were introduced in order to develop individual plans, and to support self-initiation for change. The action-plans aimed at clarifying personal goals, and make them concrete, describe how to achieve the goals, identify barriers, and describe how to overcome barriers. When action-plans were introduced at the course "Practice nurses and Type 2 diabetes", it was emphasised that the goals had to be perceived as personally important by the person compiling the action-plan. Hence, it was stressed that the health care provider should ask the question *why* people would like to work with a given action-plan

Current treatment recommendations of type 2 diabetes

In order to enhance the nurses' competences to provide clear rationales for advice given, and to provide effective options for change, course day 1 included an update on current treatment recommendations of type 2 diabetes [11]. Furthermore, course day 4 included a workshop aiming to promote discussions among the nurses on how to provide relevant information to the people with type 2 diabetes about the disease and the treatment recommendations. The starting point of the workshop discussion was the patient-worksheet "Your plans to change your way of life".

Implementation of the course content in daily practice

Between the course afternoons, we encouraged the participating nurses to fill in worksheets specifically designed for the nurses' own potential changing process. These worksheets were counterpart to the patient-worksheets, but contended issues regarding implementation of the course content in daily practice. On course day 3, we encouraged the nurses to enact a consultation with a person with type 2 diabetes from their own practice.

On course day 3 and 4, half of the time was spent on exchanging experiences from implementing the course content in daily practice, perceived barriers, and feasible actions.

The aim of the ten month-visit in the practice from one of the course-teachers was to discuss the application of the course content in daily practice.

Changed behaviour with regard to autonomy support was self-rated by the nurses 11 months after the core intervention by a mailed questionnaire.

The outcomes

The outcomes are on patient level. The primary outcomes are: 1) well-being in relation to living with diabetes, which will be measured by Problem Areas in Diabetes scale (PAID), and general well-being measured by the subscale mental health from SF-12v2, and 2) HbA1c(%)- and cholesterol (mmol/l) levels. PAID is a 20-item measure of emotional adjustment to life with diabetes. Construct validity of PAID had been investigated in American, Dutch and Swedish contexts [24-27]. HbA1c and serum-cholesterol are frequently used outcome measures as proxy indicators for prognose in diabetes [28]. HbA1c is a measure of the average plasma glucose concentration over the past two months. Serum-cholesterol is a measure of the level of lipids in the blood, and elevated levels are risk factors of cardiovascular diseases.

Secondary outcomes are 1) perceived autonomy support, 2) type of motivation, and 3) perceived competence regarding living with type 2 diabetes. Based on SDT, Health Care Climate Questionnaire (HCCQ), Perceived Competence for Diabetes Care (PCD) and Treatment Self-Regulation Questionnaire (TSRQ) were developed in order to measure perceived autonomy support, type of motivation, and perceived competence regarding living with type 2 diabetes. TSRQ was validated with regard to construct [29], and in several SDT-studies, structural equation modeling-analyses had offered an appropriate means for confirming construct validity [5-8]. The scales, mentioned, had been translated into Danish following the standardised procedure [18].

Data

The patient-perceived data will be obtained by a mailed questionnaire, and HbA1c- and cholesterol measurements will be retrieved from the laboratory database in the region. The data from the databases and the questionnaire data will be connected by the unique civil registry number assigned to all Danish citizens. Furthermore, the data will be merged with social data (education, ethnicity, cohabitation) from Statistic Denmark.

Statistical analyses

The primary analyses will be based on the intention-to-treat principle. As baseline value, the average value of the values measured within the last 12 months before course start will be used. The follow up-value will be the average value of the values measured during the 6 months following 15 months after the core intervention. Effectiveness of the intervention on HbA1c and cholesterol levels will be estimated by the differences between the diabetes populations in the intervention practices and the control practices with regard to 1) mean follow up-HbA1c and cholesterol, and 2) proportions with HbA1c≥8%, and proportions with Total cholesterol ≥5 mmol/l. The differences will be adjusted for baseline values. Differences in the proportions of the diabetes populations with measurements performed will also be assessed. Effectiveness of the intervention on score for PAID, SF-12(mental health), HCCQ, TSRQ and PCSD will be assessed by the difference between the diabetes populations in the intervention practices compared to the control practices 16 months after the core intervention.

Differences in the primary outcomes will be adjusted for age, gender, ethnicity, educational-level, and redeemed diabetes medication in the baseline-period. The analyses will be based on mixed models with including practice as a random factor in order to adjust for correlation within practices. Per-protocol analyses, including practices confirming changed behaviour and people with diabetes confirming participation in a nurse-led diabetes consultation, will be performed. Furthermore, subgroup analyses will be performed with regard to age, gender, and educational-level.

Sample size and power

It was estimated that with a minimum of 30 practices, a difference on 0.5% in mean HbA1c could be detected with a power on 90% under the assumption that 50% of the diabetes population participated in a nurse-consultation.

Ethical approval

The study will be conducted according to the Helsinki Declaration. The Danish Research Ethics Committee assessed the trial not to be a biomedical intervention, and because the intervention was addressed the nurses, informed content of the diabetes population should not be obtained. The study was approved by the Danish Data Protection Agency (j.no: 2009-41-3065), and it was registered at ClinicalTrials.gov (Identifier NCT01187069).

Discussion

This paper described an intervention aiming to improve autonomy support for people with type 2 diabetes in general practice, and the methods of evaluating the impact.

Jolly K, el al. [30] and Williams G, C et al. [15] also described the training of professionals in order to achieve autonomy supportive skills. These training programmes also included communication training, updated knowledge about treatment guidelines, focus on interactive learning, and implementation.

In our study, the intervention was offering the training course in a real-world setting. The recruitment strategy was a realistic scenario for future training courses. The design of our study was highly pragmatic [31] as there was no run-in period for the participating nurses, and according to the inclusion of an unselected diabetes population. It was possible to identify an unselected diabetes population owing to the availability of the Danish registers.

A limitation is the database's inability to distinguish between types of diabetes, but the number of type 2 diabetes far exceeds the type 1 cases, and the ratio between the types will be equal distributed between the intervention - and the control group. The inclusion of the type 1 population may however, dilute the potential effect of the intervention because type 1 diabetes care is primarily delivered by out-patient clinics. The pragmatic design is challenging with respect to demonstrating the impact of the intervention. Not all the intervention practices participated in all or even in parts of the course. In the study of Williams G, C et al. [15], the health care providers were trained until the intervention could be delivered effectively. The participating practice nurses in our study might not have changed their behaviours, and the study population might not have participated in a diabetes consultation and/or changed behaviour during the time-period. HbA1c- and cholesterol measurements have the ability to change within a short time-period given changed behaviour, but the method and interval to the behaviour change vary. The decision regarding a sufficient follow-up time is therefore challenging. The SDT-based outcomes' ability to measure change is less investigated. Another challenge of demonstrating the impact of the intervention is that an allocation to a control group may have tempted the nurses in the control practices to join another training course.

The advantages of our study design are 1) the random distribution of practice characteristics in the intervention - and the control group minimising potential confounding, and 2) the high applicability of the results to real-world practice because of the high pragmatics of attitude in our study. In a real-world setting, this study will determine the impact on the motivation, HbA1c, cholesterol, and well-being of people with diabetes by offering a training course for practice-nurses in general practices, where nurses provide diabetes consultations.

Acknowledgements and funding
The authors wish to thank Lisbet Jolin-Laursen and Helle Bang Jensby, who taught at the training course. We also wish to thank the general practices participating in the study, and the persons responding to the questionnaires. The study was supported financial by The Tryg-Fund (J.nr.7597-08), UCSF Lundbeck Foundation (J.nr. FP47/2009), and The Health Insurance Foundation (J.nr.2009B068).

Author details
[1]Department of Public Health, Section for General Practice, Aarhus University, Denmark. [2]Steno Diabetes Centre, Gentofte, Denmark. [3]Department of Public Health, Section for Biostatistics, Aarhus University, Denmark.

Authors' contributions
LJ, HTM and AS tailored the intervention and designed the study. VZ contributed to the tailoring of the intervention, and MF contributed with design and analysis considerations. LJ drafted the manuscript with all authors providing critical review and final approval.

Competing interests
The authors declare that they have no competing interests.

Received: 2 September 2011 Accepted: 24 November 2011
Published: 24 November 2011

References

1. Heart Outcomes Prevention Evaluation (HOPE) Study Investigators: **Effects of ramipril on cardiovascular and microvascular outcomes in people with diabetes mellitus: results of the HOPE study and MICRO-HOPE substudy.** *Lancet* 2000, **355**:253-259.
2. Schrier RW, Estacio RO, Esler A, Mehler P: **Effects of aggressive blood pressure control in normotensive type 2 diabetic patients on albuminuria, retinopathy and strokes.** *Kidney Int* 2002, **61**:1086-1097.
3. Gaede P, Vedel P, Larsen N, Jensen GV, Parving HH, Pedersen O: **Multifactorial intervention and cardiovascular disease in patients with type 2 diabetes.** *N Engl J Med* 2003, **348**:383-393.
4. Ryan RM, Deci EL: **Self-determination theory and the facilitation of intrinsic motivation, social development, and well-being.** *Am Psychol* 2000, **55**:68-78.
5. Williams GC, Freedman ZR, Deci EL: **Supporting autonomy to motivate patients with diabetes for glucose control.** *Diabetes Care* 1998, **21**:1644-1651.
6. Williams GC, McGregor HA, Zeldman A, Freedman ZR, Deci EL: **Testing a self-determination theory process model for promoting glycemic control through diabetes self-management.** *Health Psychol* 2004, **23**:58-66.
7. Williams GC, McGregor HA, King D, Nelson CC, Glasgow RE: **Variation in perceived competence, glycemic control, and patient satisfaction: relationship to autonomy support from physicians.** *Patient Educ Couns* 2005, **57**:39-45.
8. Williams GC, Patrick H, Niemiec CP, Williams LK, Divine G, Lafata JE, *et al*: **Reducing the health risks of diabetes: how self-determination theory may help improve medication adherence and quality of life.** *Diabetes Educ* 2009, **35**:484-492.
9. Hansen DG, Rasmussen NK, Munck AP: **Practice staff's participation in preventive measures among adult patients in general practice.** *Ugeskr Laeger* 2008, **170**:2228-2231.
10. **Ministry of Health and Prevention.** *Almen praksis'rolle i fremtidens sundhedsvæsen (The role of Primary Care in the Danish health care system)* Ministry of Health and Prevention, Denmark 2008 (in Danish).
11. Danish College of General Practitioners: **Type 2 diabetes i almen praksis. Diagnose og behandling. (Type 2 diabetes in general practice. Diagnosis and treatment).** Copenhagen: Danish College of General Practitioners; 2002, (in Danish).
12. Kristensen JK, Drivsholm TB, Carstensen B, Steding-Jensen M, Green A: **Validation of methods to identify known diabetes on the basis of health registers.** *Ugeskr Laeger* 2007, **169**:1687-1692.
13. Perera R, Heneghan C, Yudkin P: **Graphical method for depicting randomised trials of complex interventions.** *BMJ* 2007, **334**:127-129.
14. Sheldon KM, Williams G, Joiner T: **Self-Determination Theory in the Clinic. Motivating physical and mental health.** USA: Yale University Press. New Haven. London; 2003.
15. Williams GC, Patrick H, Niemiec CP, Ryan RM, Deci EL, Lavigne HM: **The Smoker's Health Project: A self-determination theory intervention to facilitate maintenance of tobacco abstinence.** *Contemp Clin Trials* 2011, **32**:535-43.
16. Aspegren K: **BEME Guide No. 2: Teaching and learning communication skills in medicine - a review with quality grading of articles.** *Med Teach* 1999, **21**:563-70.
17. Williams GC, Levesque C, Zeldman A, Wright S, Deci EL: **Health care practitioners' motivation for tobacco-dependence counseling.** *Health Educ Res* 2003, **18**:538-553.
18. Zoffmann V, Lauritzen T: **Guided self-determination improves life skills with type 1 diabetes and A1C in randomized controlled trial.** *Patient Educ Couns* 2006, **64**:78-86.
19. Zoffmann V, Kirkevold M: **Relationships and their potential for change developed in difficult type 1 diabetes.** *Qual Health Res* 2007, **17**:625-638.
20. Clabby J, O'Connor R: **Teaching learners to use mirroring: rapport lessons from neurolinguistic programming.** *Fam Med* 2004, **36**:541-543.
21. Gordon T, Kragh B: **Forældreuddannelse: problemer, konflikter, løsninger. ("Parent education: problems, conflicts, problem solving").** Denmark, Valby: Borgen; 1999, (in Danish).
22. Steinberg JM: **Aktivt verdivalg ("Active value choice").** Oslo: Aventura Forlag; 1986, (in Norwegian).

23. Maindal HT, Kirkevold M, Sandbaek A, Lauritzen T: **Lifting the lid of the "black intervention box" - the systematic development of an action competence programme for people with screen-detected dysglycaemia.** *BMC Health Serv Res* 2010, **10**:114.
24. Welch GW, Jacobson AM, Polonsky W: **The Problem Areas in Diabetes Scale. An evaluation of its clinical utility.** *Diabetes Care* 1997, **20**:760-766.
25. Amsberg S, Wredling R, Lins PE, Adamson U, Johansson UB: **The psychometric properties of the Swedish version of the Problem Areas in Diabetes Scale (Swe-PAID-20): scale development.** *Int J Nurs Stud* 2008, **45**:1319-1328.
26. El Achhab Y, Nejjari C, Chikri M, Lyoussi B: **Disease-specific health-related quality of life instruments among adults diabetic: A systematic review.** *Diabetes Res Clin Pract* 2008, **80**:171-184.
27. Eigenmann CA, Colagiuri R, Skinner TC, Trevena L: **Are current psychometric tools suitable for measuring outcomes of diabetes education?** *Diabet Med* 2009, **26**:425-436.
28. Renders Carry M, Valk Gerlof D, Griffin Simon J, Wagner E, van Eijk Jacques T, Assendelft Willem JJ: **Interventions to improve the management of diabetes mellitus in primary care, outpatient and community settings.** *Cochrane Database of Systematic Reviews 2000* 2000, **4**.
29. Levesque CS, Williams GC, Elliot D, Pickering MA, Bodenhamer B, Finley PJ: **Validating the theoretical structure of the Treatment Self-Regulation Questionnaire (TSRQ) across three different health behaviors.** *Health Educ Res* 2007, **22**:691-702.
30. Jolly K, Duda JL, Daley A, Eves FF, Mutrie N, Ntoumanis N, *et al*: **Evaluation of a standard provision versus an autonomy promotive exercise referral programme: rationale and study design.** *BMC Public Health* 2009, **9**:176.
31. Zwarenstein M, Treweek S, Gagnier JJ, Altman DG, Tunis S, Haynes B, *et al*: **Improving the reporting of pragmatic trials: an extension of the CONSORT statement.** *BMJ* 2008, **337**:2390.

Pre-publication history

The pre-publication history for this paper can be accessed here:
http://www.biomedcentral.com/1471-2296/12/130/prepub

doi:10.1186/1471-2296-12-130
Cite this article as: Juul *et al.*: A cluster randomised pragmatic trial applying Self-determination theory to type 2 diabetes care in general practice. *BMC Family Practice* 2011 **12**:130.

Submit your next manuscript to BioMed Central and take full advantage of:

- **Convenient online submission**
- **Thorough peer review**
- **No space constraints or color figure charges**
- **Immediate publication on acceptance**
- **Inclusion in PubMed, CAS, Scopus and Google Scholar**
- **Research which is freely available for redistribution**

Submit your manuscript at
www.biomedcentral.com/submit

BioMed Central
