# Supplementary material for: Effectiveness of a Training Course for General Practice Nurses in Motivation Support in Type 2 Diabetes Care: A Cluster-Randomised Trial
Source: PLoS One. 2014 May 5;9(5):e96683. doi: 10.1371/journal.pone.0096683 (PMC4010512; doi:10.1371/journal.pone.0096683)
Supplement: Protocol S2 — The first draft. (DOC) [file pone.0096683.s004.doc]

Project:

A proactive nurse-led intervention to

persons with type 2 diabetes in

general practice.

A randomised intervention study

By Lise Juul^1^

Head supervisor: Annelli Sandbaek^1^

Supervisor: Helle Terkildsen Maindal^1^

Supervisor: Vibeke Zoffmann^2^

Project group: Torsten Lauritzen^1^

Morten Frydenberg^1^

^1^Section for General Practice, Department of Public Health, Aarhus University, ^2^ Steno Diabetes Centre, Gentofte

Correspondence: Lise Juul, Section for General Practice, Department of Public Health, Aarhus University, Vennelyst Boulevard 6, st, 8000 Århus C, Denmark

Email:lise.juul@alm.au.dk

**Background**

As in other western countries, type 2 diabetes is an escalating health care problem in Denmark^1,2^. Type 2 diabetes often leads to micro- and macrovascular complications, and growing evidence suggests that an intensive multifactorial treatment including polypharmacy can prevent or delay the development of these complications^3-5^. Despite this, the treatment recommendations are often not followed; either because the physicians do not recommend the treatment, or because the people with type 2 diabetes (referred to as patients in the following) do not adhere to the treatment recommendations^6,7^. The treatment recommendations often imply radical lifestyle changes and polypharmacy, which is demanding for the patients and is life-long. It has proved difficult to relate to and act in relation to a disease that you do not feel before the complications arise^8^.

The Chronic Care Model ^9^, which the Danish Health Board has chosen as a model for optimising chronic care, describes that interaction between a well-informed active patient and a prepared proactive health care system can improve the patient´s risk profile and prognosis. Therefore, establishing cooperation with the patient with the aim of optimal treatment and improved self-management is an important professional health care challenge. Focused communication, focus on the patient´s individual challenges, use of value clarifying questions and action plans are evidence-based tools to improve the patients´self-management^10-12^.

In Denmark, type 2 diabetes care is mainly provided by general practice and the extent of the care is up to the individual practice. Variations on the organisation of administrative systems, employed practice staff, education of practice staff and the content in the consultations are many. A Cochrane review on interventions aiming to improve diabetes care in primary health care concluded that nurses can play an important role in diabetes care and can deliver a large part of it, if detailed protocols are available and if the nurses receive training^13^. Based on international research, Region Denmark encourages general practitioners to delegate both existing tasks such as quarterly consultations, but also new specialised tasks in relation to chronic care and prevention in general to practice staff^14^. Therefore, knowledge about how nurses in general practice can support the patient in living appropriately with type 2 diabetes and the effect of such an intervention on the patients´ risk profile is needed.

HbA1c and serum-cholesterol are frequently used outcome measures as proxy indicators for the prognosis of type 2 diabetes^13^. HbA1c (%) is a measure of the average plasma glucose concentration over the past two months, and serum-cholesterol (mmol/l) is a measure of the level of lipids in the blood, and an elevated concentration is a risk factor for cardiovascular disease.

Educational-level is found associated with the prognosis of type 2 diabetes^15^. Thoolen B., et al. also found educational-level to be the main factor for participation in a self-management intervention for patients with screen-detected type 2 diabetes^16^. Thus, challenges exist in developing interventions that reach patients with lower educational-levels.

**Aims and hypothesis**

The aim is to implement and in a randomised controlled trial to evaluate a proactive intervention for patients with type 2 diabetes delivered by nurses in general practice with the emphasis on improving the patients´ self-management;

1. To implement a proactive intervention
2. To assess the effect of the intervention on differences in 1) clinical values, 2) performed measurements and 3) patient-perceived outcomes e.g. perceived support.
3. To assess to what extent the intervention effect is associated with educational-level, gender or age.
4. To assess whether educational-level, gender and age affect the patients with type 2 diabetes´participation in nurse-led consultations in general practice.

Hypothesis

We expected that the intervention would enhance the patients´ self-management; which would reduce their risk profile regarding complications indicated by HbA1c- and total cholesterol levels 15 months after the invitation to a proactive nurse-led consultation. We expected that the patients with type 2 diabetes regardless of educational-level, gender or age would benefit from the intervention.

**Methods**

*Intervention, design and study population*

The intervention will be described and developed according to the MRC-model^17^. The underlying theories will be inter alia the Chronic Care Model^9^, The method Guided self-determination^10,11^ and Diffusion of innovations^18^. It will include a training course for nurses on the implementation of tools for the joint planning of care (between patient and nurse). The content in the training course will include knowledge about type 2 diabetes and treatment recommendations, the Chronic Care Model and the method Guided Self-determination. Furthermore, it will include communication based on adult learning-principles; case-based. The aim of implementing the tools is to guide a focused patient-centered communication. They consist of two reflection-sheets from the method Guided self-determination and an action plan from the Chronic Care Model. The focus is on the patients´self-perceived problems living with diabetes and at which stadium; the patient is regarding willingness to change behaviour. The action plans aim at clarifying personal goals, making them tangible, identifying barriers, and describing how to overcome these. To prevent type III failure, a nurse with practical experience of the tools will be included in the intervention to support the nurses in the intervention practices (”linkage agent”^18)^. The development of the intervention components and their feasibility will be pilot-tested in minimum two general practices before intervention start.

The effect of the intervention will be evaluated by a cluster randomised study with 40 general practices from the former county of Aarhus, and approximately 2500 patients with type 2 diabetes , > 40 years old, identified by linkage with the Aarhus Diabetes Database.

All the general practices from the former county of Aarhus with nurse-led diabetes consultations will be invited to participate in the study. For larger enrollment than necessary, a random selection will be performed. The included practices will be randomised to a control and an intervention group. Twenty control practices will continue as usual, whereas the intervention will be offered to 20 intervention practices. All the patients will be followed 15 months after the nurses have completed the two first course days.

The process will be assessed by follow-up of a fixed cohort (the patients in the intervention arm, approx.. 1250 persons)

*Power calculation*

It has been estimated that with a minimum of 30 practices, a difference of 0.5% in mean HbA1c can be detected with a power of 90 % under the assumption that 50% of the diabetes population from the Aarhus Diabetes Database will participate in a nurse-led consultation.

*Outcome*

The primary outcomes are changes in 1) HbA1c (%) and 2) serum-cholesterol (mmol/l). The last identified value 12 months before index-date will be used as baseline, and the 15 month value will be identified by interpolation between the last value before and first value within three months after index-date ( 1^st^- date after nurse participation on second course day) + 15 months.

Secondary outcomes are 1) The proportion of patients with HbA1c and serum-cholesterol measurements 9 – 15 months after index-date and 2) the patients self-perceived outcomes, e.g perceived support.

Patients who come to at least one nurse-led consultation will be identified. Use of work-sheets will be assessed.

*Data*

Data will be collected via registers, nurse registration and patient questionnaires.

Major revisions of this first protocol to the current protocol:

Visits to Danish general practices with nurse-led diabetes consultations during the intervention development process indicated that invitation of the diabetes population to nurse-led diabetes consultations would not be feasible at the current time. Therefore, we decided to improve the content of the nurse-led diabetes consultations under the current conditions. The visits also gave the impression that checklist-driven counselling with a lot of information and advice giving was present in the nurse-led diabetes consultations. We therefore chose the Self-determination Theory as the underlying theory for our intervention. This theory proposes a patient-centred approach with the main focus on the underlying reasons for motivation for actions and it also emphasises the importance of adequate information from health care providers. The Self-determination Theory was well-supported by observational studies and an explanatory trial in a Danish diabetes outpatient clinic; but documentation on the effect of applying the theory in the current framework of type 2 diabetes care in general practice was lacking. The patient self-perceived outcomes were specified according to the Self-determination Theory in the intervention developing phase. The current protocol ; the one described in the published paper is approved by the Danish Data Protection Agency.

**Referencer**

1. Borch-Johnsen K. Type 2 diabetes. Prevention of a public disease and its consequences. Ugeskr.Laeger 2004; 166:1316-1320.
2. Glumer C, Jorgensen T, Borch-Johnsen K, et al. Prevalences of diabetes and impaired glucose regulation in a Danish population: the Inter99 study. Diabetes Care 2003; 26:2335-2340.
3. Effects of ramipril on cardiovascular and microvascular outcomes in people with diabetes mellitus: results of the HOPE study and MICRO-HOPE substudy. Heart Outcomes Prevention Evaluation Study Investigators. Lancet 2000; 355:253-259.
4. Schrier RW, Estacio RO, Esler A, et al. Effects of aggressive blood pressure control in normotensive type 2 diabetic patients on albuminuria, retinopathy and strokes. Kidney Int. 2002; 61:1086-1097.
5. Gaede P, Vedel P, Larsen N, et al. Multifactorial intervention and cardiovascular disease in patients with type 2 diabetes. N Engl J Med 2003; 348:383-393.
6. Vermeire E, Wens J, Van Royen P, et al. Interventions for improving adherence to treatment recommendations in people with type 2 diabetes mellitus. *Cochrane Database of systematic Reviews 2005,* Issue 2. Art. No.: CD003638. DOI: 0.1002/14651858.CD003638.pub2.
7. Kristensen JK, Lauritzen T. Polypharmacological treatment of type 2 diabetes in daily clinical practice. A registry-based study in the county of Aarhus. Ugeskr Laeger 2002;164:5250-5253.
8. Wiuff MB. Intensiv polyfarmakologisk behandling af patienter med type 2-diabetes i daglig klinisk praksis. En medicinsk teknologivurdering. København: Sundhedsstyrelsen og Dansk Sundhedsinstitut, 2007.
9. Wagner EH. Chronic disease management: what will it take to improve care for chronic illness? Eff Clin Pract 1998;1:2-4.
10. Zoffmann V, Lauritzen T. Guided self-determination improves life skills with type 1 diabetes and A1C in randomized controlled trial. Patient Educ.Couns 2006;64:78-86.
11. Zoffmann V, Harder I, Kirkevold M. A person-centered communication and reflection model: sharing decision-making in chronic care. Qual Health Res 2008;18:670-685.
12. Handley M, MacGregor K, Schillinger D, Sharifi C, Wong S, Bodenheimer T. Using action plans to help primary care patients adopt healthy behaviors: a descriptive study. J Am Board Fam Med 2006;19:224-231.
13. Renders CM, Valk GD, Griffin S, et al. Interventions to improve the management of diabetes mellitus in primary care, outpatient and community settings. *Cochrane Database of Systematic Reviews* 2000, Issue 4. Art. No.: CD001481. DOI: 10.1002/14651858.CD001481.
14. Danske Regioner. Almen praksis i Fremtidens Sundhedsvæsen, 2007; [http://www.regioner.dk/PolitikOgHoldninger/Sundhed%20og%20sygehuse/Almen%20Praksis%20i%20Fremtidens%20Sundhedsv-ae-sen.aspx](http://www.regioner.dk/PolitikOgHoldninger/Sundhed og sygehuse/Almen Praksis i Fremtidens Sundhedsv-ae-sen.aspx).
15. Espelt A, Borrell C, Roskam AJ, Rodriguez-Sanz M, Stirbu I, Dalmau-Bueno A, et al. Socioeconomic inequalities in diabetes mellitus across Europe at the beginning of the 21st century. Diabetologia 2008;9.
16. Thoolen B, de Ridder D, Bensing J, Gorter K, Rutten G. Who participates in diabetes self-management interventions?: Issues of recruitment and retainment. Diabetes Educ 2007;33:465-474.
17. Campbell M, Fitzpatrick R, Haines A, et al. Framework for design and evaluation of complex interventions to improve health. BMJ 2000;321:694-696.
18. Oldenburg B, Parcel GS. Diffusion of innovations. In: Glanz K, Rimer BK, Lewis FM. Health behaviour and health education. San Francisco: Jossey-Bass; 2002. p. 312-334.
